# Calculation of Short-Term Creep of Concrete Using Fractional Viscoelastic Model

**DOI:** 10.3390/ma16124274

**Published:** 2023-06-08

**Authors:** Shengqi Mei, Xufeng Li, Xingju Wang, Xiaodong Liu

**Affiliations:** 1 State Key Laboratory of Mechanical Behavior and System Safety of Traffic Engineering Structures, Shijiazhuang Tiedao University, Shijiazhuang 050043, China; 2 Key Laboratory of Roads and Railway Engineering Safety Control of Ministry of Education, Shijiazhuang Tiedao University, Shijiazhuang 050043, China; 3 School of Civil Engineering, Shijiazhuang Tiedao University, Shijiazhuang 050043, China; 1202201058@student.stdu.edu.cn (X.L.); 1202201197@student.stdu.edu.cn (X.L.); 4 School of Transportation, Shijiazhuang Tiedao University, Shijiazhuang 050043, China; wangxingju@stdu.edu.cn

**Keywords:** concrete creep, ultra-short load duration time, viscoelasticity, fractional order calculus

## Abstract

The study of short-term creep is essential for understanding the concrete creep process and deformation under alternating stress. Researchers are concentrating on the nano- and micron-scale creep of cement pastes. In the latest RILEM creep database, short-term concrete creep data at hourly or minutely levels are still rare and scarce. In order to describe the short-term creep and creep-recovery behavior of concrete specimens more accurately, the short-term creep and creep-recovery experiments were carried out firstly. The load-holding time varied from 60 s to 1800 s. Secondly, the accuracy of current creep models (B4, B4s, MC2010, and ACI209) in predicting the short-term creep of concrete was compared. It was discovered that the B4, B4s, and MC2010 models all overestimate concrete’s short-term creep, and the ACI model does the opposite. Thirdly, the applicability of the fractional-order-derivative viscoelastic model (with a derivative order between 0 and 1) in the calculation of the short-term creep and creep recovery of concrete is investigated. The calculation results show that the fractional-order derivatives are more suitable for analyzing the static viscoelastic deformation of concrete while the classical viscoelastic model requires a large number of parameters. Therefore, a modified fractional-order viscoelastic model is proposed considering the residual deformation characteristics of concrete after unloading, and the values of the model parameters under different conditions are given with the experimental data.

## 1. Introduction

Concrete creep can lead to excessive structural deformation, even up to 1~3 times the elastic deformation during the loading phase [1]. Due to creep and durability, the annual repairing cost of highways and bridges in the U.S. is about $78.8 billion [2]. Multiple hazards cause direct losses (physical loss) and indirect losses (impacts over time). Although there are already a number of ways to assess these losses [3,4], it is still a research challenge to exclude the aging of the concrete material itself as well as the coupled effects of concrete shrinkage and external environmental changes that occur during long-term testing [5,6].

Woolson [7] first discovered the flow of concrete under pressure in 1905. Glanville and Thomas [8,9] attributed the phenomenon of concrete creep to the viscous flow of cement paste. A large number of experimental studies [10], computational analyses [11], and mechanistic explanations [12] have been carried out for concrete creep. However, as Bažant stated, the concrete creep has not been fully grasped [13]. The mechanism of concrete creep includes consolidation theory, plastic flow, the action of free water in the pore space, microcrack expansion, etc. [14].

Hassan et al. [15] compared the test results of a three-minute- and two-day-long load duration of concrete creep and found that concrete had almost no aging behavior during the three-minute test time, while concrete with the two-day test time had microstructural aging. C-S-H generated by cement hydration is one of the main factors contributing to the complex viscoelastic behavior of concrete. When the load-holding time is short, the deformation growth of concrete mainly comes from the load action; this phenomenon is more responsive to the viscoelastic nature of concrete. Regarding the creep behavior during the load-holding phase, Vandamme and Ulm [16] tested different C-S-H density zones by the nanoindentation technique and concluded that the creep may be due to the rearrangement of nanoscale particles near the limiting stacking density. Alizadeh et al. [17] analyzed the three-point bending stress relaxation of synthetic C-S-H materials and cement slurry tests and demonstrated that the presence of interlayer water has an important effect on the viscoelastic properties of C-S-H. The mechanism of the C-S-H viscoelastic properties was explained by the following reasons: a. Effect of C-S-H crystallinity: The difference between the crystallinity of C-S-H in the cement paste and the synthesized C-S-H leads to different stress relaxation; b. Stress redistribution: The pressure generated by the load under the holding load gradually transitions from all materials to the unhydrated cement and calcium hydroxide fraction; and c. The relative sliding of the microstructure within the C-S-H leads to a more pronounced stress relaxation in the synthetic C-S-H.

Gan et al. [18] presented an experimental investigation on the short-term creep recovery of cement paste at the micrometric length scale. It is found that cement pastes show high recovery ratios even when subjected to very high stress. A good agreement is found between the results predicted using the linear superposition principle and the experimental results except for the measured non-linear creep of samples with a 0.4 w/c ratio. Creep tests on concrete specimens indicate a nonlinear dependence of the creep strain rate on the acting stress for high stress levels. Dummer et al. [19] confirmed the nonlinear dependence of the creep strain rate on the acting stress for high sustained acting compressive stress for both sealed and drying conditions by the conducted creep tests. In addition, the degree of nonlinearity in the creep tests on sealed specimens was found to be significantly higher than in the creep tests on drying specimens.

Ferretti and Leo [20] showed that in axial compression experiments, under constant load, the time dependence of displacement is strictly related to the growth of microcracks with microseismic analysis experiments, and creep is a material property that has no significant effect on macroscopic behavior. Ma et al. [21] established a creep model considering the composition of mesoscale materials based on the DEM and Burgers model. The contact network, force-chain probability distribution, and meso-fabric anisotropy were assessed to explain the creep mechanism of concrete. Xu et al. [22] established a concrete creep model considering the ITZ’s viscoelasticity to predict the creep behavior of concrete, and the ITZ effect on concrete creep performance was studied. The ITZ contribution to concrete creep increased with the loading age.

The large number of factors influencing concrete creep and the large dispersion of test data lead to deviations in the prediction results of different creep models. Short-term concrete test data can be used to reduce the uncertainty of model parameters [23]. The effectiveness and ease of calibration of creep models using short-term creep data was mentioned by the ACI committee, and the uncertainty could be significantly reduced by data within a one-month test time [24]. However, even in the latest RILEM creep database, short-term concrete creep data at hourly or minutely levels are still rare and scarce [13]. Su et al. [25] realized a short-term load-holding test for concrete materials at different stress levels and performed a computational analysis using existing creep models. It was found that existing creep models are not yet effective in predicting the short-term creep of concrete at a minute level. 

By fitting the parameters of the models to the experimental results of laboratory testing, the evolution of the long-term deformation of the real structures can be relatively well-estimated. Aili et al. [26] presented a fully coupled approach to predict delayed strains of concrete. Baronet et al. [27] developed a novel two-scale method (TSM) which combines an as-short-as-possible uniaxial compression creep test and rapid microindentation creep tests to predict the two parameters of the logarithmic basic creep function. In addition, the method was shown to be practical and accurate.

Classical viscoelastic models require a variety of spring and dashpot elements for specific applications, and lead to the complexity of parameter identification. Fractional-order viscoelastic models have been continuously applied in the analysis of the creep cracking of plain concrete [28], viscoelastic strain of asphalt concrete [29], viscoelasticity of metallic materials [30] and creep recovery of asphalt materials [31] due to their ability to describe the creep behavior of materials more accurately and with fewer parameters to be identified.

Calculation models of the short-term creep and creep recovery of concrete at the macro scale within the minutes duration time are still rare. In order to describe the short-term creep and creep-recovery behavior of concrete specimens more accurately, the static viscoelastic behavior of concrete materials is analyzed based on short-term creep experiments. The prediction effectiveness of the existing concrete creep models (i.e., B4, B4s, MC2010, and ACI209R-92 models), the classical viscoelastic model and the fractional-order viscoelastic model for creep under short-term load holding is analyzed. A modified fractional-order viscoelastic model is established considering the residual deformation characteristics. The scope of the research is limited to normal concrete, without considering the influence of manufacture-sand, admixtures, etc.

## 2. Materials and Methods

### 2.1. Materials

**Figure 1:**
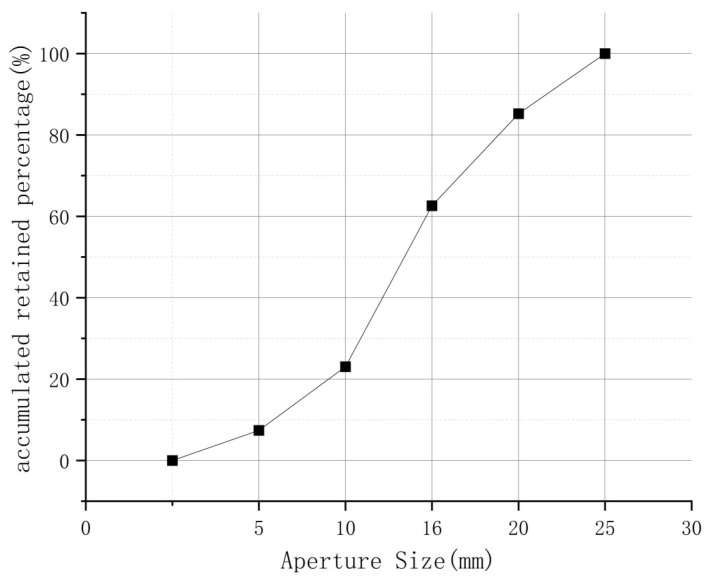
Grading curve of coarse aggregates.

C40 and C50 grade concrete was used in this study. The cement is PO42.5R ordinary silicate cement, fine aggregate is sand, and coarse aggregate is crushed stone. The aggregate gradation curve is shown in Figure 1, which meets the requirements for continuous gradation from 5 to 25 mm. The chemical composition of cement is shown in Table 1. The mix proportions for C40 grade concrete is cement:fly ash:water:fine aggregate:coarse aggregate:water reducer = 1:0.19:0.43:2.01:2.69:0.023. The mix proportions for C50 grade concrete is cement:fly ash:water:fine aggregate:coarse aggregate:water reducer = 1:0.17:0.38:1.52:2.28:0.026. 

Fresh concrete was mixed following China GB/T50082-2009 standard using a mixer and cast in moulds. After demoulding, the samples were cured under steam conditions at temperature 20 °C for 28 days. The specific specimen sizes are cubic 100 mm × 100 mm × 100 mm for compressive strength test, and 100 mm × 100 mm × 300 mm for compressive strength test and modulus of elasticity test. The specimen size for short-term creep test is also prismatic 100 mm × 100 mm × 300 mm. 

The mechanical properties, i.e., compressive strength and modulus of elasticity of concrete, are shown in Table 2. The compressive strength of C40 concrete is 42 MPa and the mean modulus of elasticity is 32.39 GPa; the compressive strength of C50 concrete is 48.5 MPa and the modulus of elasticity is 34.58 GPa. 

### 2.2. Test Preparation

During the test, the concrete deformation data were output using a dynamic strain gauge. To ensure the accuracy of the test data, resistance strain gauges were pasted on four sides of the specimens. The accuracy of the strain gauge was 0.5 με. The sampling frequency was set as 25 Hz considering the strain acquisition during loading and unloading phases. 

The specimens were aligned by laser before the test to ensure the axial force of the concrete specimens. The specimens were precompressed with a preload of 5 kN before the formal test, and the dynamic strain gauge could display the four sets of strain data in real time. By observing the real-time data of precompression of strain gauges, the eccentricity was analyzed and the position of the specimens was adjusted in time. In addition, the average value of the strains measured on the four sides was used as the overall strain of the specimen. If the maximum value of the 4 groups of strain data differs from the minimum value by more than 15%, the group of data needs to be rejected.

### 2.3. Creep and Creep-Recovery Test

The loading force of C40 grade concrete specimens is 100 kN. The loading force of C50 grade concrete specimens is 150 kN. The test conditions are shown in Table 3. For example, T40-10-60 means the target load of C40 concrete is 100 kN, corresponding to the concrete stress of 10 MPa, and the load-holding time is 60 s.

**Figure 2:**
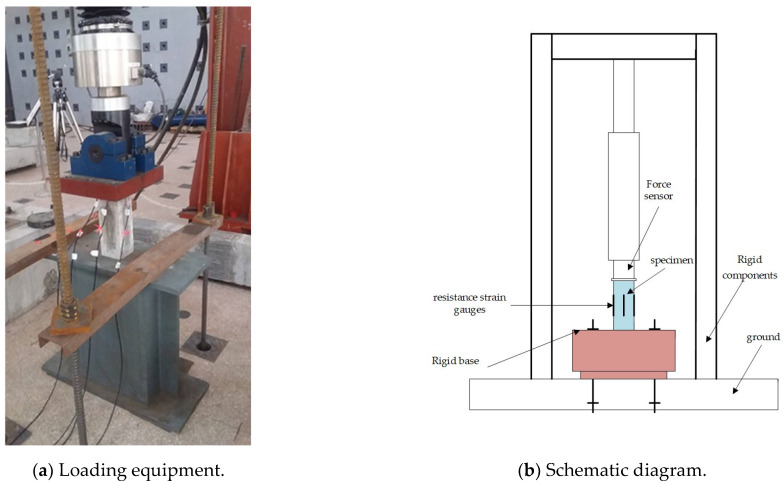
Loading equipment and schematic diagram.

The creep test device recommended in China standard GB/T50082-2009 [32] needs to be loaded by twisting screws. In this paper, the tests of short-term creep and creep recovery of concrete materials are carried out by using a hydraulic servo device, which can realize instantaneous unloading. The loading equipment was a 20 T electro-hydraulic servo multifunctional testing machine from the National Engineering Laboratory of Bridge Structure Safety Technology of Ministry of Transportation of China, with a displacement accuracy of better than ±0.5% of the full scale and a load accuracy of better than ±0.5% of the full scale. The test piece is placed on a rigid base and fixed on the anchor in the test site. The loading equipment and schematic diagram are shown in Figure 2. Before the installation of the test device, the upper and lower surfaces of the base are polished to ensure the flatness of the surface. When the initial installation of the base is completed, a laser is used to draw a line between the base and the indenter of the test machine to determine the location of the center point.

**Figure 3:**
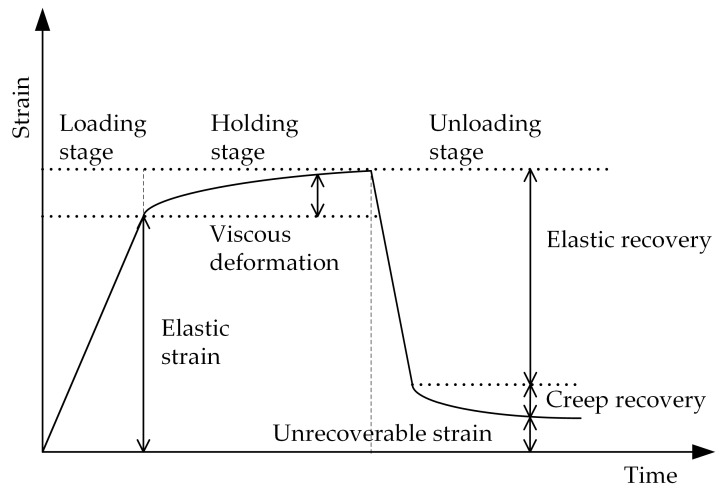
Schematic diagram of deformation during the test.

It is now generally accepted that concrete is in the elastic state when the stress is less than 40% of the compression strength. As shown in Figure 3, during the test, elastic strain is referred to as the strain during loading stage and creep strain is referred to as the strain during load-holding stage. It is noticed that the creep strain include creep during the loading phase. Elastic recovery describes the recovery of strain during the unloading stage, while hysteresis recovery describes the recovery of strain after the load has been completely removed, i.e., creep recovery. The non-recoverable deformation is called plastic deformation or residual deformation.

## 3. Results

### 3.1. Creep Strain Evolution

**Figure 4:**
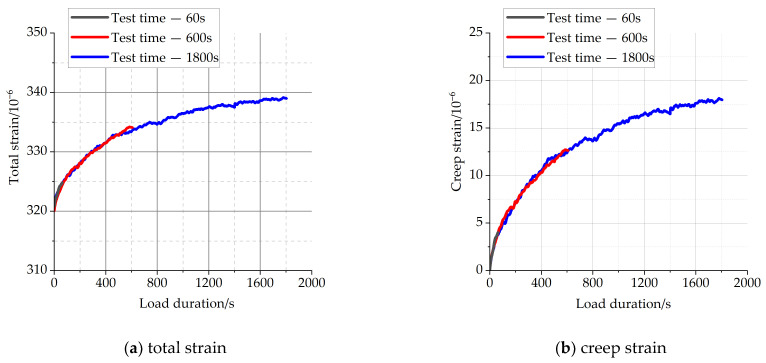
The strain of C40 concrete with different load duration.

**Figure 5:**
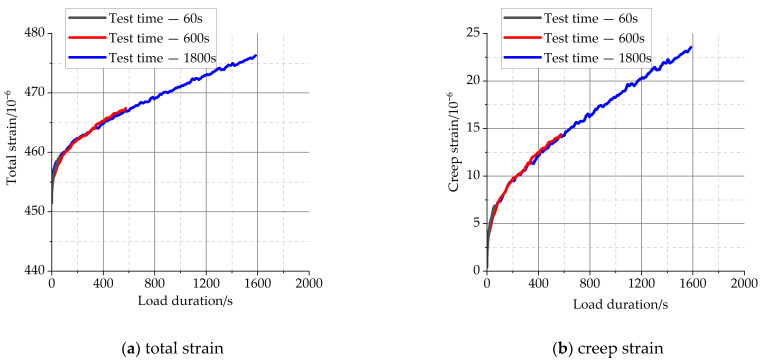
The strain of C50 concrete with different load duration.

Figure 4 and Figure 5 show the total strains and creep strains of concrete specimens at different load-holding times of 60 s, 600 s, and 1800 s, respectively. The position of the starting point of each total strain curve in the figure represents the axial strain of each specimen at the completion of loading.

From Figure 4 and Figure 5, it can be observed that the total strains of concrete specimens continue to increase with time throughout the load-holding process, and the growth rate is faster in the early stage for the chosen value of load during the load-holding stage. There is a substantial overlap between the strain curves for the shorter holding time conditions and the strain curves for the longer holding time conditions. For the short holding time conditions, concrete creep is still in accordance with the superposition principle. The specific creep values during load-holding stage are shown in Table 4, and the creep strains show a non-linear growth trend with time.

### 3.2. Creep-Recovery Evolution

**Figure 6:**
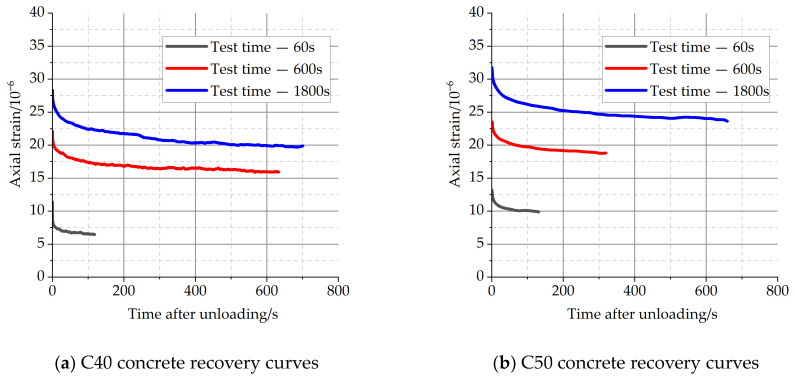
Hysteretic recovery curves of concrete specimens after different load duration.

The recovery of deformation is a good way to distinguish the elastic deformation, the aging deformation, and the non-recoverable deformation during the loading and load-holding stages. After unloading, concrete generally produces an elastic recovery of deformation. As shown in Figure 3, there is a partial slow recovery of deformation after elastic recovery. Figure 6 illustrates the hysteresis recovery curves of concrete for a variety of load-holding time conditions. It is possible that the unrecoverable deformation can be attributed to microscopic damage to the concrete during the loading phase, as well as viscous flow.

As can be seen from Figure 6, the recovery rate of concrete after unloading gradually slows down with time until it stabilizes. Because of the small creep strain during the load-holding stage, the recovery of deformation for specimens with short holding times typically tends to settle fast after unloading. Long holding times for specimens result in long deformation stabilization recovery times. When the hysteresis recovery reached stability, the total value of the hysteresis recovery strain for each condition was statistically analyzed, as shown in Table 5.

As can be seen in Table 5, the total value of hysteresis deformation recovery increased by about 1.2 and 1.3 times for C40 and C50 concrete, respectively, as the load-holding time increased from 60 s to 1800 s. For the concrete specimens, the hysteresis deformation recovery after unloading increased with the increase of stress level.

There will be some residual deformation when concrete is discharged after the load-holding stage. Table 6 gives the residual strains of concrete specimens for different load-holding times.

From Table 6, it can be seen that the residual deformation after unloading increases significantly with the increase of the holding time. The residual deformation increased by 1.5 times and 2.1 times for C40 concrete and about 0.9 times and 1.4 times for C50 concrete when the load-holding time increased from 60 s to 600 s and 1800 s, respectively. The result shows that residual deformation also needs to be considered as a limit state within the structural creep analysis. However, based on the viscoelastic model, this is not possible to be considered.

## 4. Comparisons between Experimental Results and Predictions

### 4.1. Existing Computational Models

**Figure 7:**
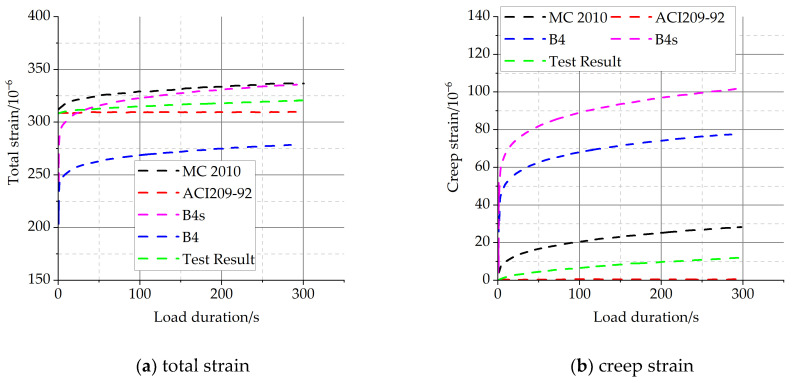
Comparison of the creep experimental results and prediction results by creep models.

The predictions of short-term concrete creep using exiting creep models (i.e., B4, B4s, MC2010, and ACI209-92 model) are presented in Figure 7; the unit of total and creep strain is με. The test results are collected from the tests at a holding time of 300 s. The parameter inputs in each creep model are determined according to the test results.

It can be seen from Table 7 that the prediction results of the B4s, MC2010, and ACI209-92 models are closer to the experimental test results. The ACI209-92 model underestimated the deformation of C40 concrete under a short-term load duration, and both the B4s and MC2010 models overestimated the deformation. The reason may be that the B4s and MC2010 models set the concrete strength as the main parameter in the specific calculation. The calculated results using the B4 model tend to approach the experimental results as time increases. Figure 7b shows the creep strains in the load-holding phase. The results show that the ACI209-92 model underestimates the creep deformation of concrete in the loading stage, and other models overestimate the creep deformation of concrete to varying degrees (B4s model > B4 model > MC2010 model).

The Boltzmann superposition principle states that when multiple loads act together, the final deformation is related to each load action separately; i.e., each load increase or decrease step is independent and can be mathematically superimposed on each other [33]. The corresponding stresses are $\sigma \left(t\right)=\sum_{i=0}^{n}\Delta \sigma \left(\tau_{i}\right),\tau_{n}=t$ and the strains are $\varepsilon \left(t\right)≅\sum_{i=0}^{n}\Delta \sigma \left(\tau_{i}\right)\varphi \left(t,\tau \right)$. According to the superposition principle approach, some researchers have used a concrete creep model for the computational analysis of creep recovery [34]. The recovery of deformation after unloading is considered equivalent to the superposition of creep occurring from forces acting in the opposite direction.

**Figure 8:**
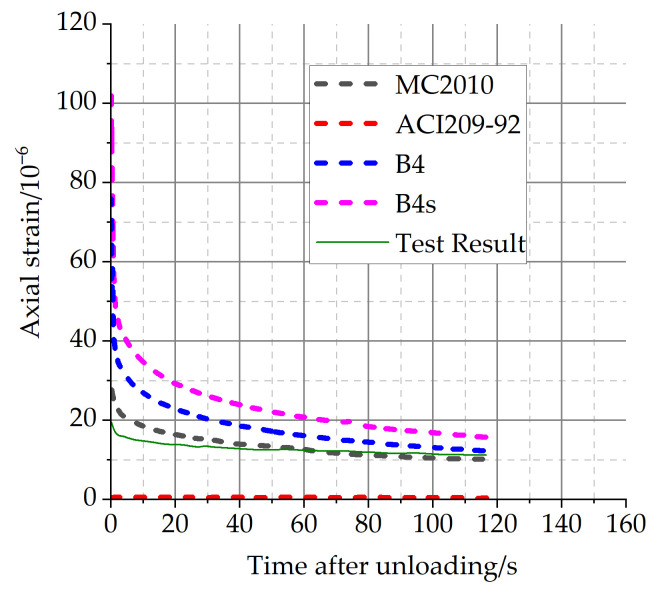
Comparison between calculation results and creep-recovery test data of C40 concrete.

According to the calculation method of the superposition principle, the calculation results of the creep-recovery characteristics by using four creep models are analyzed and compared with the test results of C40 concrete, as shown in Figure 8.

As can be seen in Figure 8, the calculation results using the B4, B4s, and MC2010 models are all relatively close to the test deformation, except for the ACI209-92 model. 

It is interesting to note that existing creep prediction models overestimate the prediction of short-term creep, but the prediction of creep recovery is very comparable. The mechanisms of creep recovery and creep may be different, and the law of deformation development is not the same. On the other hand, the process of creep recovery is not disturbed by the external load again, and the value of creep recovery is relatively small.

### 4.2. Short-Term Creep Prediction Based on Viscoelastic Model

The deformation of concrete under static forces and the recovery of deformation have a distinct time-dependent property, or viscoelastic quality. To describe this property, a time-dependent model needs to be constructed. The viscoelastic model has been used to describe the creep behavior of concrete. In this section, the static viscoelastic behavior of concrete at different stress levels is analyzed by using the classical viscoelastic model and the fractional viscoelastic model.

#### 4.2.1. Classical Viscoelastic Model

The stress–strain relationship of the Kelvin model is:

(1) $$\sigma \left(t\right)=E\varepsilon \left(t\right)+\mu \frac{d\varepsilon \left(t\right)}{dt}$$

The principal relationship of the Maxwell model is shown as:

(2) $$\sigma \left(t\right)+\frac{\mu }{E}\cdot \frac{d\sigma \left(t\right)}{dt}=\mu \frac{d\varepsilon \left(t\right)}{dt}$$

The creep compliance of the Maxwell model is:

(3) $$J\left(t\right)=\frac{1}{E}+\frac{1}{\mu }\cdot t$$

Nineteen viscoelastic models were summarized by Neville et al. [35] and it was concluded that, when the number of elements is too large, some ill-posed problems are introduced. Therefore, simplifying the number of elements and parameters of the viscoelastic model not only facilitates the calculation process, but also helps to clarify the physical meaning of the model parameters [36].

#### 4.2.2. Fractional-Order Viscoelastic Model

Reiner [37] believes that the viscoelastic model of concrete should satisfy the following conditions: (a). When the load starts to act, instantaneous strain occurs; (b). When the stress is less than a certain value, no creep occurs; and (c). When the stress is greater than a certain value and remains as a constant, the strain grows gradually with time and the rate of change of strain decreases gradually with time. The above conditions describe the creep of concrete behavior characterized by the viscoelastic model, but do not define the recovery of deformation after unloading. 

In the classical viscoelastic model of integer order, a single dashpot element describes only a single hysteresis mechanism. Schiesel et al. [38] demonstrated by operations in the frequency domain that the mechanical model of a fractional-order viscous element can be viewed as a tree fractal network model consisting of a series of springs and dashpot elements, which can better reflect the viscoelasticity of the material over the entire load duration.

If the stress–strain relationship of the spring element in the classical viscoelastic model is expressed as $\sigma \left(t\right)=Ed^{0}\varepsilon \left(t\right)/dt^{0}$, the stress–strain relationship of the dashpot element is expressed as $\sigma \left(t\right)=\mu d^{1}\varepsilon \left(t\right)/dt^{1}$; then, the stress–strain relationship of the viscoelastic material between them can be characterized by a fractional order calculus: $\sigma \left(t\right)∼d^{\alpha }\varepsilon \left(t\right)/dt^{\alpha },(0<\alpha <1)$.

**Figure 9:**
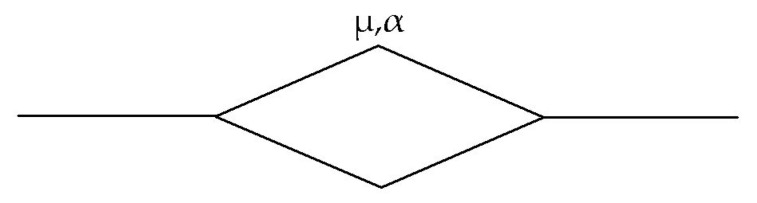
Fractional element.

The dashpot element in the viscoelastic model is replaced with a fractional-order viscous element, as shown in Figure 9.

The stress–strain relationship of the fractional element is:

(4) $$\sigma =\mu \cdot \frac{d^{\alpha }\varepsilon \left(t\right)}{dt^{\alpha }},\alpha \in \left(0,1\right)$$

When α=0, the equation can be written as $\sigma =\mu \cdot d^{0}\varepsilon \left(t\right)/d^{0}t$; when α=1, the equation can be written as $\sigma =\mu \cdot d^{1}\varepsilon \left(t\right)/d^{1}t$.

The fractional-order derivative is defined as:

(5) $$\frac{d^{\alpha }f\left(x\right)}{dx^{\alpha }}=\frac{1}{\Gamma \left(1-\alpha \right)}\cdot \int_{0}^{x}\frac{f^{\prime }\left(y\right)}{{\left(x-y\right)}^{\alpha }}dy,\alpha \in \left(0,1\right)$$

where Γ is Gamma function, $\Gamma \left(z\right)=\int_{0}^{\infty }e^{-t}\cdot t^{z-1}dt,\;\mathrm{and}\;\Gamma \left(0\right)=\infty ,\Gamma \left(1\right)=1$.

From Equations (4) and (5), the creep compliance of the fractional-order viscous element is:

(6) $$J\left(t\right)=\frac{1}{\mu }\cdot \frac{t^{\alpha }}{\Gamma \left(1+\alpha \right)}$$

**Figure 10:**
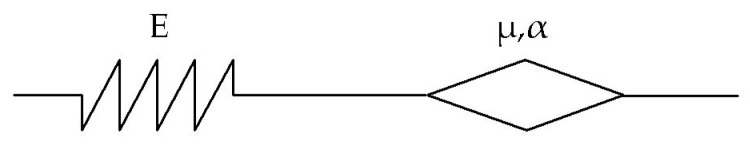
Diagram of fractional Maxwell model.

To further analyze the applicability of fractional-order calculus in describing the static viscoelastic properties of concrete, fractional-order elements are used to replace the viscous elements in the Maxwell model to form a fractional-order Maxwell model, as shown in Figure 10.

Then, the creep compliance of the fractional-order Maxwell model is:

(7) $$J\left(t\right)=\frac{1}{E}+\frac{1}{\mu }\cdot \frac{t^{\alpha }}{\Gamma \left(1+\alpha \right)}$$

where E is the elasticity parameter, μ is the viscosity coefficient, and α is the fractional order of the derivative.

**Figure 11:**
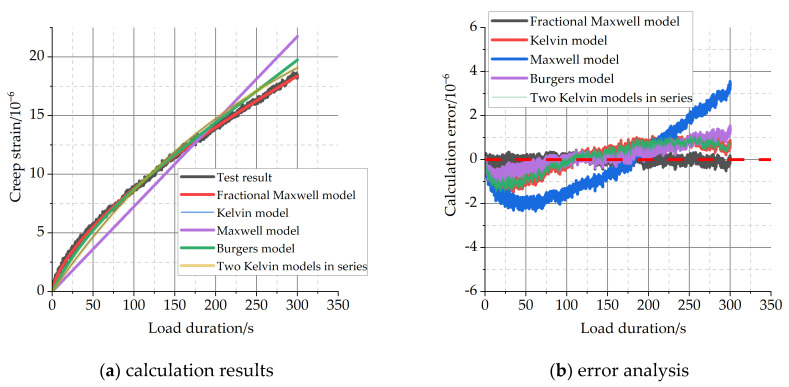
Calculation results of creep by different viscoelastic models.

The effects of the classical viscoelastic model and the fractional-order viscoelastic model on the calculation of short-term creep are compared, as shown in Figure 11. The classical viscoelastic models are the Maxwell model, Kelvin model, and Burgers model formed by a series connection of the Maxwell model and Kelvin model, and two Kelvin models in series. The fitting method uses the Levenberg–Marquardt algorithm [39]. Figure 11a shows the analysis of the calculated results of short-term creep, and Figure 11b shows the error of the calculated results and the test results.

From Figure 11, it is clear that the fractional-order derivative model can simulate the short-term creep of concrete material well compared with the above four viscoelastic models. 

**Figure 12:**
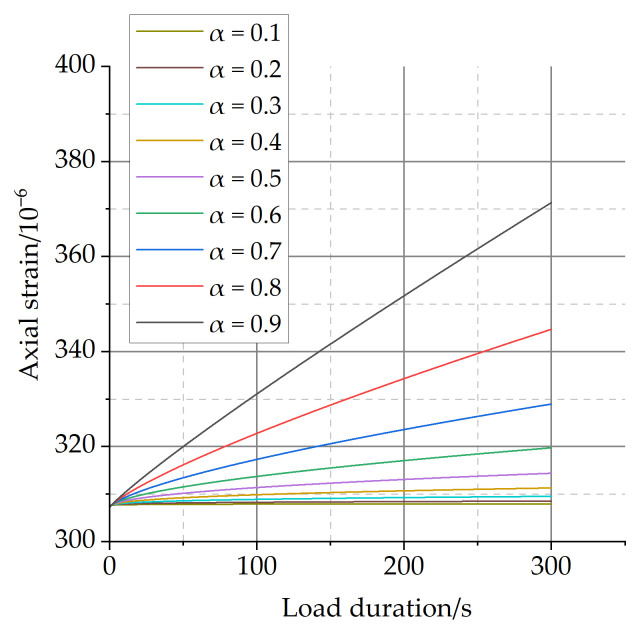
Parameter analysis of fractional Maxwell model.

The characteristics of the fractional-order Maxwell model are analyzed with the fractional-order derivative as the variable, as shown in Figure 12.

From Figure 12, it can be seen that when the fractional-order derivative tends to 1, the deformation in the load-holding phase grows significantly and its deformation characteristics tend to be the ideal viscous behavior, and when the fractional-order derivative tends to 0, the deformation in the load-holding phase decreases significantly and its deformation characteristics tend to be the ideal elastic behavior. 

The deformation characteristics of different viscoelastic materials can be well-reflected by the change of fractional-order derivatives in the fractional-order Maxwell model. Therefore, the prediction accuracy of the three-parameter fractional-order Maxwell model for the short-term creep of ordinary concrete can exceed that of the classic viscoelastic model in Figure 12.

#### 4.2.3. Modified Fractional-Order Viscoelastic Model

**Figure 13:**
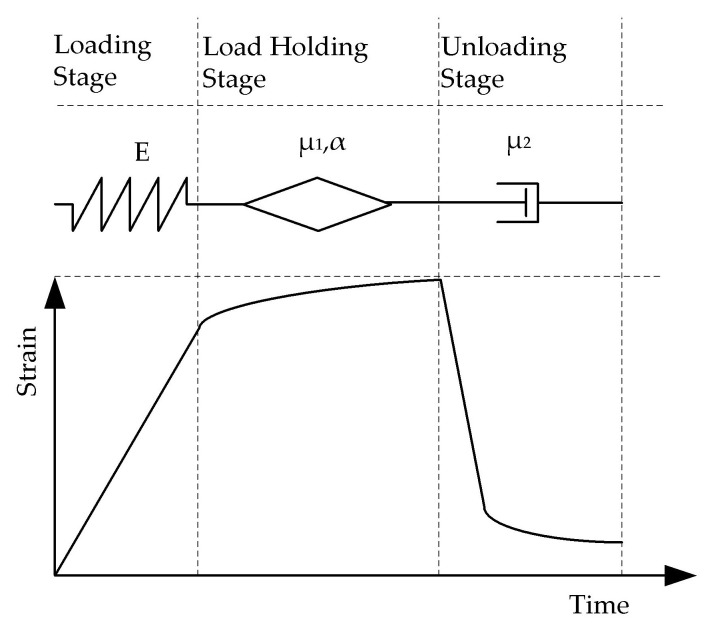
Modified fractional Maxwell model.

In order to analyze the hysteresis deformation recovery characteristics of concrete materials, a viscous element is added to the fractional-order Maxwell model in this section, and the new model established is shown in Figure 13.

The stress–strain relationship for the viscous element added in the model can be expressed as $\sigma \left(t\right)=\mu_{2}\dot{\varepsilon }$, where $\sigma \left(t\right)$ is the stress corresponding to the element, $\mu_{2}$ is the coefficient of viscosity, and $\dot{\varepsilon }$ is the strain rate corresponding to the element. In the holding phase, this part of the deformation is approximately considered to grow linearly with time, reflecting the development of the irrecoverable part of the deformation process with time. In the post-unloading deformation phase, this part of the deformation is the residual deformation.

The expression for calculating the strain in the load-holding phase of the modified fractional-order Maxwell model is:

(8) $$\varepsilon \left(t\right)=\left(\frac{1}{E}+\frac{1}{\mu_{1}}\cdot \frac{t^{\alpha }}{\Gamma \left(1+\alpha \right)}+\frac{t}{\mu_{2}}\right)\cdot \sigma_{0}$$

**Figure 14:**
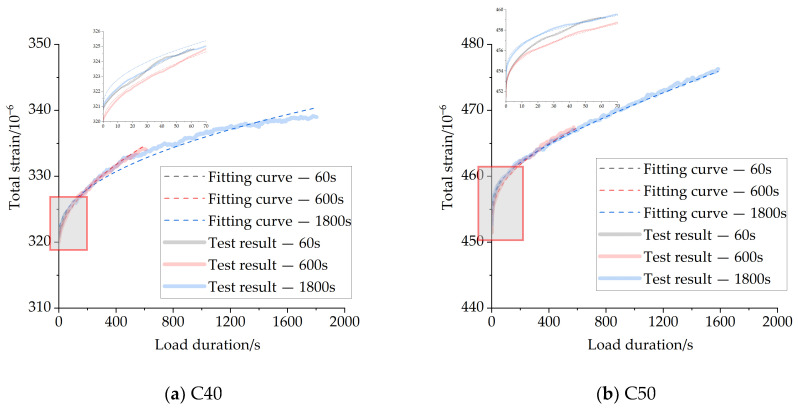
Fitting curve of creep strain under different load duration.

The results of the creep tests for C40 and C50 concrete with different load-holding times were analyzed using the modified fractional-order viscoelastic model, and the same Levenberg–Marquardt algorithm was used for the fitting method. The test fitting results are shown in Figure 14, and the identified model parameters are shown in Table 8.

From the results of the data analysis in Figure 14, it can be seen that the modified fractional-order Maxwell model can analyze well the deformation behavior of both strength grades of concrete in the short-term load-holding phase, and the *R*^2^ for different conditions are above 0.9, as shown in Table 8.

There is a certain error between the calculated results for the 1800 s holding time and the measured data in Figure 14, which is within 5%. This may be due to the complexity of the concrete material components and internal microstructure, which leads to the deformation law under different holding times is not strictly consistent. In this section, only three main parameters are used in the modified fractional-order Maxwell model to reflect the deformation behavior in the load-holding phase in order to ensure the simplicity of the calculation process, which may also lead to some deviations between the calculated results and the measured data.

From the identification results of the model parameters in Table 8, it can be seen that the derivative order of the fractional-order components of the model tends to decrease as the load-holding time increases for both C40 concrete and C50 concrete. The reason for this phenomenon is due to the fact that, for the chosen value of load during the load-holding stage, concrete material creep develops faster in the early stage and the creep growth rate decreases gradually with the increase of time. This law of deformation development is reflected in the viscoelastic model; that is, when the early viscoelastic deformation is large, the later growth trend decreases, and the corresponding derivative order gradually decreases.

### 4.3. Discussion

For concrete, the analysis of long-term creep behavior may be inaccurate if short-term test data are used directly, and a link between short-term and long-term creep needs to be established. At the same time, the above data illustrate the advantage of the fractional-order viscoelastic model for the static viscoelastic property analysis of materials. Deformation behavior at different stages can be achieved by adjusting the fractional-order derivatives.

## 5. Conclusions

The deformation analysis of concrete specimens was carried out based on short-term creep/recovery tests of concrete. It was found that the residual deformation trends and the deformation trends in the load-holding phase were not significantly the same. 

Four creep prediction models (B4 model, B4s model, MC2010 model, and ACI209-92 model) were used to compare and analyze the concrete deformation test data under short-term load-holding conditions. The calculation results show that all the models overestimate the short-term creep of concrete except the ACI209-92 model which underestimates the short-term creep of concrete.

Then, the short-term creep behavior of concrete was analyzed based on the classical viscoelastic theory and fractional-order derivative viscoelastic theory. It was found that the prediction accuracy of the fractional-order Maxwell model for short-term creep behavior was higher than other models and required fewer parameters.

A modified fractional-order Maxwell model with the addition of viscous elements is proposed. A comparison analysis with the experimental data shows that the proposed model can reflect the creep-recovery behavior of concrete well. The effects of the modified model are analyzed for different load-holding durations and the values of the model parameters are given for each condition in combination with the experimental data.

## Figures and Tables

**Table 1:** Chemical composition of cement (%).

SiO_2_	Al_2_O_3_	Fe_2_O_3_	CaO	MgO	SO_3_	Na_2_O	f-CaO	Loss	CL
21.28	4.73	3.41	62.49	2.53	2.83	0.56	0.72	1.76	0.011

**Table 2:** Mechanical properties of concrete materials.

Strength Grade	Specimen Number	Cube Compressive Strength/MPa	Prismatic Compressive Strength/MPa	Modulus of Elasticity/GPa
C40	1	45	41.9	32.58
	2	46.3	42.0	33.12
	3	45.3	42.2	31.46
	Average value	45.5	42	32.39
C50	1	52.3	47.7	34.25
	2	51.5	48.6	34.86
	3	51.2	49.2	34.63
	Average value	51.67	48.5	34.58

**Table 3:** Test conditions.

Test Series	Concrete Strength Grade	Holding Load (kN)	Stress (MPa)	Stress Ratio	Load Duration (s)
T40-10-60	C40	100	10	0.24	60
T40-10-600	C40	100	10	0.24	600
T40-10-1800	C40	100	10	0.24	1800
T50-15-60	C50	150	15	0.31	60
T50-15-600	C50	150	15	0.31	600
T50-15-1800	C50	150	15	0.31	1800

**Table 4:** Creep strains under different load duration (unit: με).

Load-Holding Time (s)	C40 Concrete	C50 Concrete
60	4.02	6.91
600	12.66	14.40
1800	17.98	23.56

**Table 5:** Total hysteresis recovery strains under different load duration times (unit: με).

Load-Holding Time	C40 Concrete	C50 Concrete
60 s	3.86	4.24
600 s	6.19	7.82
1800 s	8.43	9.96

**Table 6:** The residual strain under different load duration (unit: με).

Load-Holding Time	C40 Concrete	C50 Concrete
60 s	6.47	9.89
600 s	15.93	18.89
1800 s	19.88	23.80

**Table 7:** Strain value at 300 s (unit: με).

	Test Result	B4	B4s	MC2010	ACI209-92
Strain value	321	279	336	337	309
error	-	0.131	0.046	0.049	0.035

**Table 8:** Fitting parameters of concrete at load duration stage.

Load Conditions	$1/\mu_{1}$	α	$1/\mu_{2}$	*R* ^2^
T40-10-60	0.0247	0.66	6.50 × 10^−14^	0.990
T40-10-600	0.0410	0.54	8.53 × 10^−19^	0.998
T40-10-1800	0.0546	0.46	2.03 × 10^−19^	0.988
T50-15-60	0.108	0.35	1.85 × 10^−4^	0.998
T50-15-600	0.110	0.31	2.83 × 10^−4^	0.999
T50-15-1800	0.130	0.25	4.01 × 10^−4^	0.999

## Data Availability

All data included in this study are available upon request by contacting the corresponding author.
